# Supplementary material for: Content-rich biological network constructed by mining PubMed abstracts
Source: BMC Bioinformatics. 2004 Oct 8;5:147. doi: 10.1186/1471-2105-5-147 (PMC528731; doi:10.1186/1471-2105-5-147)
Supplement: Additional File 2 — The original results of the above study (non-essential files are deleted to keep the file size under the limit set by BMC bioinformatics). [file 1471-2105-5-147-S2.bz2 › chilibotAdditionalFile2/dip10/700ID10869074E18033/html/MIND_MINC.html]

 


 **MIND** and **MINC** 
  
Found 51 abstracts in PubMed, retrieved 10.  
 

 What does Google say? 
 PDF only 
| .edu only 

---

**Interactive relationship** (e.g. stimulation, inhibition, etc)

**Neutral relationship**- iii both  **MinC**  and MinE can be recruited to  **MinD** .ATP decorated vesicles.  Ref: 12533449 J Bacteriol, 2003
- Also, it is argued that binding of  **MinD**  to the membrane activates  **MinC** .  Ref: 12486056 J Bacteriol, 2003
- We observed that  **MinD**  dimerized in the presence of ATP and interacted with  **MinC** .  Ref: 12486056 J Bacteriol, 2003
- In the presence of a phospholipid bilayer,  **MinD**  bound to the bilayer and recruited  **MinC**  in an ATP dependent manner.  Ref: 12486056 J Bacteriol, 2003
- A conserved sequence at the C terminus of  **MinD**  is required for binding to the membrane and targeting  **MinC**  to the septum.  Ref: 12519187 Mol Microbiol, 2003
- These results suggest that MinE induces a conformational change in  **MinD**  bound to the bilayer that results in the release of  **MinC** .  Ref: 12486056 J Bacteriol, 2003
- In contrast,  **MinC**  was unable to displace MinE bound to the  **MinD**  bilayer complex.  Ref: 12486056 J Bacteriol, 2003
- **MinC**  dynamics involves a membrane association dissociation cycle that is driven by the activities of the  **MinD**  ATPase and the MinE topological specificity factor, which themselves undergo coupled oscillatory localization cycles.  Ref: 12533449 J Bacteriol, 2003
- In the presence of ATP  **MinD**  binds  **MinC**  and goes to the membrane the ATPase is not stimulated and the complex is stable.  Ref: 12675792 Mol Microbiol, 2003

**Non-interactive relationship** (e.g. studied together, co-existance, homology, etc.)

- The Min system comprises  **MinD**  and  **MinC** , which form an inhibitor complex and, in Bacillus subtilis, DivIVA, which ensures that division is inhibited only in the polar regions.  Ref: 12492852 Mol Microbiol, 2003
- The results also indicate that MinE stimulated dissociation of  **MinC**  from the  **MinC**   **MinD** .ATP membrane complex can, and may, occur prior to hydrolysis of the nucleotide.  Ref: 12533449 J Bacteriol, 2003
- and v MinE stimulates dissociation of  **MinC**  from  **MinD** .ATP membrane complexes, even when ATP hydrolysis is blocked.  Ref: 12533449 J Bacteriol, 2003
- Introduction of a negatively charged amino acid decreased membrane binding of  **MinD**  and its ability to activate  **MinC** .  Ref: 12867440 J Bacteriol, 2003
- Addition of MinE to the MinCD bilayer complex resulted in release of both  **MinC**  and  **MinD** .  Ref: 12486056 J Bacteriol, 2003
- Accurate positioning of the division septum at the equator of Escherichia coli cells requires a rapid oscillation of  **MinD**  ATPase between the polar halves of the cell membrane, together with the division inhibitor  **MinC** , under MinE control.  Ref: 12482939 Proc Natl Acad Sci U S A, 2002
- The release of  **MinC**  did not require ATP hydrolysis, indicating that MinE could displace  **MinC**  from the  **MinD**  bilayer complex.  Ref: 12486056 J Bacteriol, 2003
- **MinD**  localizes to the cytoplasmic face of the inner membrane in Escherichia coli, and its association with the inner membrane is a prerequisite for membrane recruitment of the septation inhibitor  **MinC** .  Ref: 12424340 Proc Natl Acad Sci U S A, 2002
- The Min system of Escherichia coli directs cell division to the mid cell by a mechanism that involves the dynamic localization of all of its three constituent proteins,  **MinC** ,  **MinD**  and MinE.  Ref: 12368455 Microbiology, 2002
- Recruitment of  **MinC** , an inhibitor of Z ring formation, to the membrane in Escherichia coli role of  **MinD**  and MinE.  Ref: 12486056 J Bacteriol, 2003
